# Protection provided by vaccination, booster doses and previous infection against covid-19 infection, hospitalisation or death over time in Czechia

**DOI:** 10.1371/journal.pone.0270801

**Published:** 2022-07-08

**Authors:** Luděk Berec, Martin Šmíd, Lenka Přibylová, Ondřej Májek, Tomáš Pavlík, Jiří Jarkovský, Milan Zajíček, Jakub Weiner, Tamara Barusová, Jan Trnka

**Affiliations:** 1 Centre for Mathematical Biology, Institute of Mathematics, Faculty of Science, University of South Bohemia, České Budějovice, Czech Republic; 2 Department of Ecology, Czech Academy of Sciences, Biology Centre, Institute of Entomology, České Budějovice, Czech Republic; 3 Centre for Modelling of Biological and Social Processes, Praha, Czech Republic; 4 Czech Academy of Sciences, Institute of Information Theory and Automation, Praha, Czech Republic; 5 Department of Mathematics and Statistics, Faculty of Science, Masaryk University, Brno, Czech Republic; 6 Institute of Biostatistics and Analyses, Faculty of Medicine, Masaryk University, Brno, Czech Republic; 7 Institute of Health Information and Statistics of the Czech Republic, Praha, Czech Republic; 8 Siesta Labs, Praha, Czech Republic; 9 First Faculty of Medicine, Charles University, Praha, Czech Republic; 10 Department of Statistical Modelling, Czech Academy of Sciences, Institute of Computer Science, Praha, Czech Republic; 11 Department of Biochemistry, Cell and Molecular Praha, Czech Republic; University of Hail, SAUDI ARABIA

## Abstract

Studies demonstrating the waning of post-vaccination and post-infection immunity against covid-19 generally analyzed a limited range of vaccines or subsets of populations. Using Czech national health data from the beginning of the covid-19 pandemic till November 20, 2021 we estimated the risks of reinfection, breakthrough infection, hospitalization and death by a Cox regression adjusted for sex, age, vaccine type and vaccination status. Vaccine effectiveness against infection declined from 87% at 0-2 months after the second dose to 53% at 7-8 months for BNT162b2 vaccine, from 90% at 0-2 months to 65% at 7-8 months for mRNA-1273, and from 83% at 0-2 months to 55% at 5-6 months for the ChAdOx1-S. Effectiveness against hospitalization and deaths declined by about 15% and 10%, respectively, during the first 6-8 months. Boosters (third dose) returned the protection to the levels observed shortly after dose 2. In unvaccinated, previously infected individuals the protection against infection declined from 97% after 2 months to 72% at 18 months. Our results confirm the waning of vaccination-induced immunity against infection and a smaller decline in the protection against hospitalization and death. Boosting restores the original vaccine effectiveness. Post-infection immunity also decreases over time.

## Introduction

The availability of vaccines brought about a breakthrough in the fight against the coronavirus disease 2019 (covid-19) worldwide. In the light of the economic and social costs already caused by covid-19, and the widespread aversion to any serious limitations of people’s daily lives due to lockdowns, vaccination is undoubtedly a key tool for the containment of the pandemic and for the limiting of its devastating impact on lives and health of people around the globe. Proving in the clinical studies and the weeks and months of their real-world application their high effectiveness against SARS-CoV-2 infection, symptomatic covid-19 illness, need of a hospital admission, the probability of severe symptoms, and death [1–4], their continued impact now starts to be challenged by an increasing proportion of breakthrough infections and illnesses in fully vaccinated individuals [5, 6].

In the Czech Republic, vaccination started on December 27, 2020 initially with the mRNA-based vaccine BNT162b2 (Pfizer/BioNTech), followed by mRNA-1273 (Moderna), and the adenovirus-based vector vaccines ChAdOx1-S (AstraZeneca) and Ad26.COV2-S (Johnson&Johnson). The administration of booster doses then started on September 20, 2021 and was initially open to all individuals who completed their vaccination 8 months or longer ago with only BNT162b2 and mRNA-1273 as allowed boosting vaccines. The waiting period was shortened to 6 months on October 29, 2021. Until November 1, 2021 a full 100 μg dose of Moderna was being administered as a booster dose and since that day only a 50 μg dose was used. We emphasize that by complete vaccination we mean two doses of vaccine (and just one for Ad26.COV2-S) and by booster we mean the third vaccine dose.

Central Europe experienced another wave of SARS-CoV-2 infections in the autumn of 2021 despite the substantial proportion of vaccinated and/or recovered population. This wave was accompanied by a non-negligible proportion of new infections in vaccinated individuals including the need for hospital admission and, in a relatively few cases, for intensive care. The appearance of breakthrough infections, though not unexpected, has complicated the public health messaging related to the importance of vaccination and calls for a better understanding of the temporal dynamics of post-vaccination immunity in real-world settings. Post-infection immunity is another important factor determining individual risk. SARS-CoV-2 reinfections have been reported as relatively rare events, yet the post-infection immunity appears to wane, too [7–10]. None of these studies addressed longer-term dynamics of post-infection immunity and their relationship to post-vaccination immunity.

## Materials and methods

### Study population and data sources

The analyses are based on data from the Czech National Information System of Infectious Diseases (ISID), which includes records of all individuals tested positive for SARS-CoV-2 in the Czech Republic since the beginning of covid-19 pandemic [11]. This database is overseen by the Czech Ministry of Health and operated by the Institute of Health Information and Statistics of the Czech Republic. The ISID data is routinely collected in compliance with Czech legal regulations (Act on the Protection of Public Health). The Director of the Institute of Health Information and Statistics of the Czech Republic has granted that there is no need for ethical approval of the retrospective analyses presented in this paper. Among other things, the ISID database covers demographic data, dates of vaccination, including the vaccine types for each dose, and dates of infection and potential reinfection, including information on dates of hospital admission with covid-19, and death with covid-19. Additional information on deaths from any cause come from the Death Certificate System; these data are used for censoring purposes only.

In total, our dataset contains 7,428,968 valid records of vaccinated and/or SARS-CoV-2 positive persons (additional 8,834 cases lack information on sex or age and 216 other cases contain data errors, see S2 Table in the S2 File). We further excluded 16,399 persons who were recorded to die by the start of vaccination (December 26, 2020). As the source dataset consists only of those who were tested positive and/or were vaccinated, we completed the sample to the whole population such that the added subjects were neither tested positive nor vaccinated. In particular, we completed each sex-age category to the numbers reported by the Czech Statistical Office by December 31, 2020—10,701,777 inhabitants; consequently, our sample truly reflected the sex and age structure of the whole population, containing all the positive and/or vaccinated individuals. We neglected births and deaths of the added persons.

### Vaccine types and vaccination and infection dynamics

**Fig 1:**
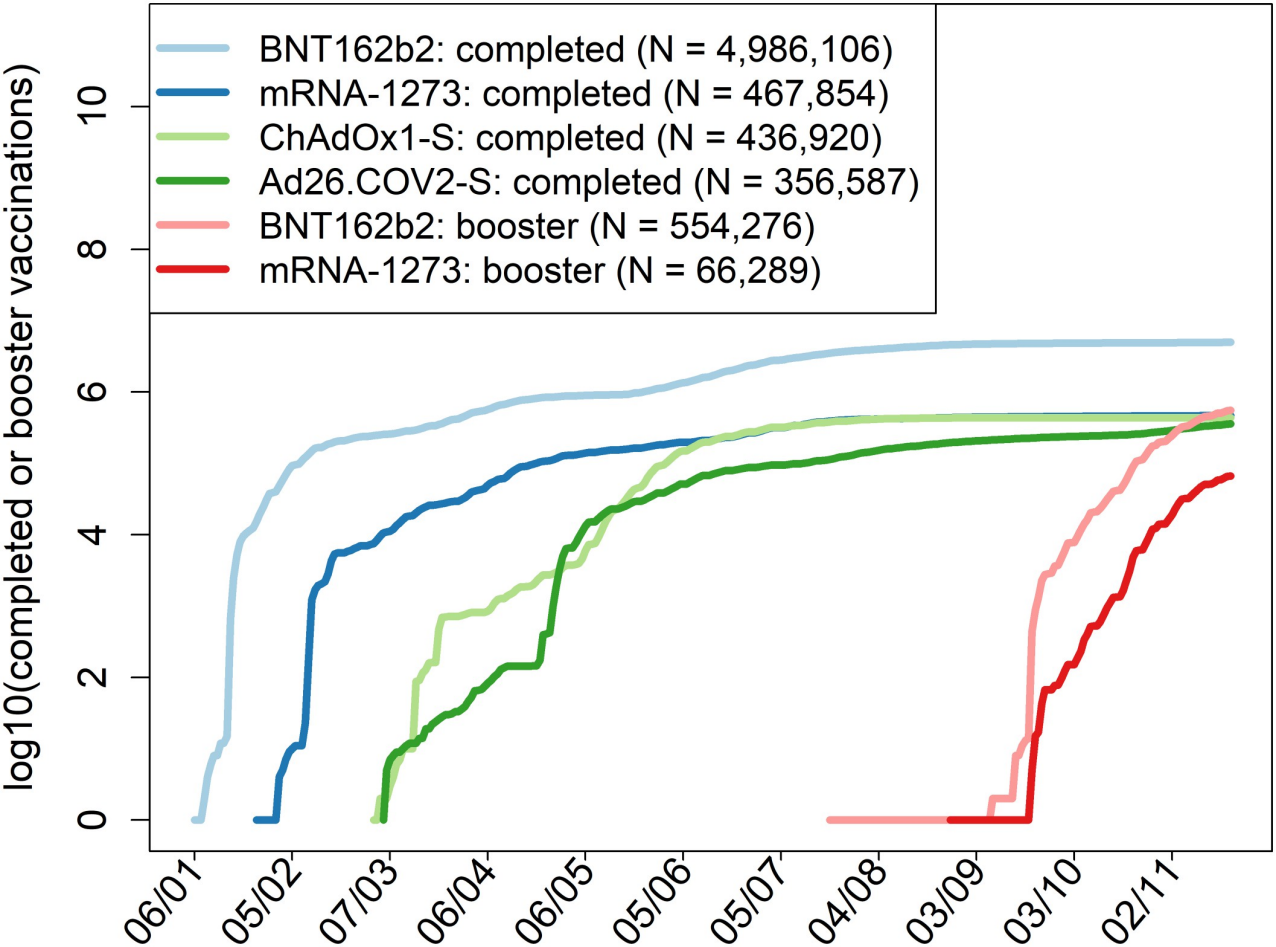
Dynamics of vaccination in the Czech Republic. Specific days in which vaccination was open to an age group or professional or other category are specified in S1 Table in S2 File.

In the Czech Republic, all EMA-approved Covid-19 vaccines have been distributed and used. They were provided to all individuals at no cost following the Czech public health insurance system. Starting on December 27, 2020, workers in the critical infrastructure were vaccinated first, followed since January 15, 2021 by persons of age 80 and older (S1 Table in S2 File). As of November 20, 2021, the national Institute of Health Information and Statistics reported 6,287,356 individuals completing the vaccination (58.75% of the population and 67.36% of persons of age 12 years and older); see Fig 1.

The covid-19 epidemic in the Czech Republic started with the first three cases reported on March 1, 2020 and was initially fueled by Czech citizens returning from the alpine ski resorts of Italy and Austria. Since then the country saw five waves of covid-19 spread. As of November 20, 2021, 1,996,080 individuals were infected with SARS-CoV-2 virus, of which 12,894 (0.65%) were reinfected; see S1 Fig in S2 File. See also S2 and S3 Tables and the accompanying S2 and S3 Figs in S2 File for an overview of the numbers of infection-related outcomes and vaccines applied within various age cohorts.

### Statistical analysis

We separately studied three types of events: (i) SARS-CoV-2 infection defined as a PCR confirmed positive test of a person from any sample regardless of the presence of symptoms, (ii) hospital admission of a person tested positive via a PCR test (within two weeks before hospital admission and whenever during hospitalization), and (iii) death due to covid-19.

A Cox regression with time-varying covariates was applied to estimate hazard ratios (HRs) for the outcomes of interest. Analogously to [12], we used calendar time instead of time from event occurrence as the time scale. Thus, the time course of individual cases was modelled using “switching” dummy variables, corresponding to the stages of the process the subject goes through. The vaccine effectiveness is calculated by comparing hazards of the vaccinated individuals to those of the control group—those who have not been vaccinated and infected so far—individually for each vaccine type [12]. By using calendar time, we could control for changing epidemic conditions, including non-pharmaceutical measures, seasonal effects and viral variants; these phenomena can then be encompassed in the baseline hazard function. Subjects were withdrawn from the study at the time of their (covid or non-covid) death.

Time zero corresponded to the day before the start of vaccination (26 December 2020) for the analyses of vaccine-induced immunity, and the onset of epidemic in the Czech Republic for the analyses of infection-induced immunity. Moreover, we estimated how HRs of infection after vaccination depended on time after the vaccine application (adjusted for sex, age and time since the last infection), how HRs of hospital admission or death depended on time after the vaccine application (adjusted for sex and age), and how HRs of reinfection in unvaccinated individiuals depended on time since the previous infection (adjusted for sex and age). In all these cases, we estimated the vaccine effectiveness (VE, regarding a previous infection as a “vaccine”) as VE = 1 − HR [12–14].

We aggregate the time delays in two-month (61 days) periods. We consider one such period after the first dose, four periods after the second one and a single period after the booster dose. When a new dose is applied to a person, (s)he is no longer regarded to be in any period corresponding to the previous dose, but enters the first period corresponding to the new dose. In line with the Czech vaccination recognition policy, the first period corresponding to any of the first two doses starts two weeks after the dose application, while for boosters this interval is just 7 days. For the reinfections, we consider nine two-month periods.

We examine boosting effects by the mRNA vaccines BNT162b2 and mRNA-1273. Since 93% of BNT162b2 boosters is preceded by the BNT162b2 second dose and 73% of mRNA-1273 boosters is preceded by the mRNA-1273 second dose, but the vaccine type used for the first two doses does not play a role in which type is applied as the booster, we estimate the boosting effects of the mRNA vaccines both with and without individual vaccination history. Subjects with the alleged application of ChAdOx1-S (in total 176) or Ad26.COV2-S (in total 332) boosters are withdrawn from the study of booster dose effectiveness, as these records are most likely data entry errors.

To analyse a possible impact of the delta variant to breakthrough infections, we performed an alternative analysis including dummy variables indicating time period starting on July 1st, 2021 when the delta variant started to dominate in Czechia (virus.img.cas.cz/lineages). We performed three such comparisons, each concerning only age cohorts which started to be extensively vaccinated at similar time (S1 Table in S2 File); only this way could guarantee to some extent that the estimation of the delta effect would not collide with that of immunity waning.

All calculations were performed using the R software (package survival). The algorithm used to transform data from the database into the package command inputs was coded in C++. See Supporting information for details.

## Results

Since December 26, 2020 to November 20, 2021, 6,287,356 individuals received complete vaccination (58.75% of the population and 67.36% of persons of age 12 years and older). In this period a total of 1,335,055 individuals were infected, of which 96,237 (7.21%) were hospitalized and 20,809 (1.56%) died because of covid-19 (S2 Table and S2 Fig in S2 File). Among vaccinated individuals by far the largest group of 5,011,115 persons (79.7%) received BNT162b2, followed by 469,605 persons (7.47%) vaccinated with mRNA-1273, 436,575 persons (6.94%) with ChAdOx1-S and 370,061 persons (5.89%) with the one-dose Ad26.COV2-S vaccine (S3 Table and S3 Fig in S2 File). The 693,071 booster doses administered in this period comprised 617,002 doses of BNT162b2 and 76,069 doses of mRNA-1273 (S3 Table and S3 Fig in S2 File). We emphasize again that by complete or full vaccination we mean two doses of vaccine (and just one for Ad26.COV2-S) and by booster we mean the third vaccine dose.

**Fig 2:**
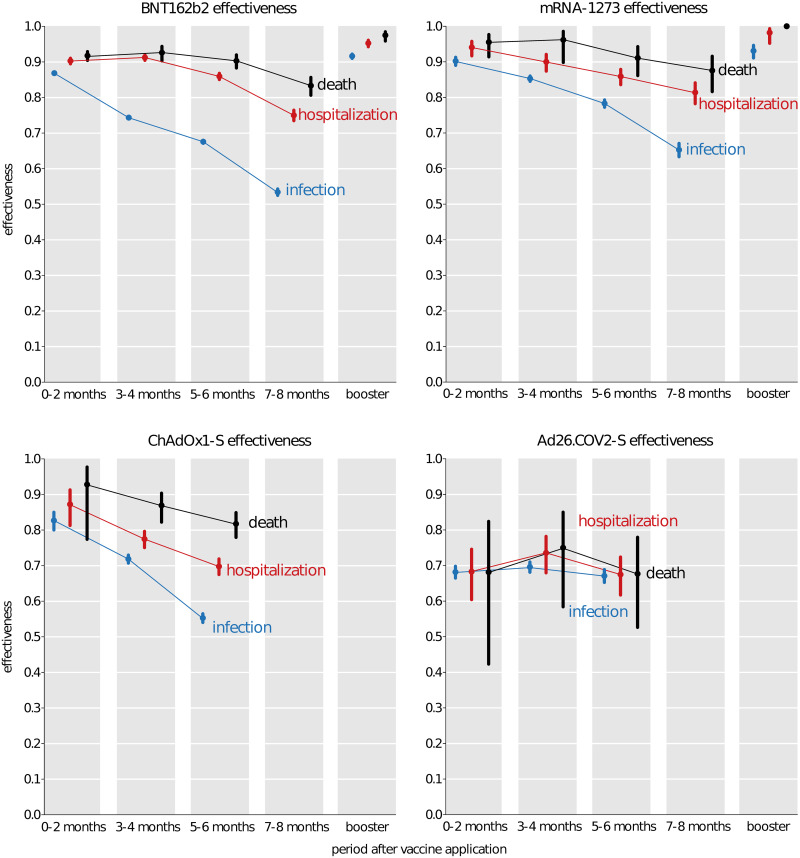
Vaccine effectiveness against infection. Vaccine-acquired immunity against infection with respect to the delay from the full vaccine application, including the effect of a booster vaccine dose.

Using a Cox regression model we estimated changes in vaccine effectiveness over time at two-month intervals (Fig 2, S4 Table in S2 File). The vaccine effectiveness against any PCR-confirmed SARS-CoV-2 infection declined for BNT162b2 from 87% (95% CI 86–87) 0–2 months after the second dose to 53% (95% CI 52–54) at 7–8 months, for mRNA-1273 from 90% (95% CI 89–91) at 0–2 months to 65% (95% CI 63–67) at 7–8 months, and for ChAdOx1-S from 83% (95% CI 80–85) at 0–2 months to 55% (95% CI 54–56) at 5–6 months. Interestingly, the estimated effectiveness for the Ad26.COV2-S vaccine (68% (95% CI 66–70) at 0–2 months and 67% (95% CI 65–69) at 5–6 months) did not seem to exhibit any significant decline over the study period but notably starts at a significantly lower effectiveness. The effectiveness estimates for ChAdOx1-S and Ad26.COV2-S at 7–8 months after the completion of vaccination exhibit very large uncertainty due to a low number of events as most people completed their vaccination with these vaccines much later, and are therefore only shown in S4 Table in S2 File.

A similar trend can be seen in the estimation of vaccine effectiveness against hospital admissions and deaths. For hospital admission, the vaccine effectiveness declined for BNT162b2 from 90% (95% CI 89–91) at 0–2 months after dose 2 to 75% (95% CI 73–76) at 7–8 months, for mRNA-1273 from 94% (95% CI 92–96) to 81% (95% CI 78–84), and for ChAdOx1-S from 87% (95% CI 81–91) at 0–2 months to 70% (95% CI 68–72) at 5–6 months (Fig 2 red curves, S4 Table in S2 File). In the case of protection from death the model estimated for BNT162b2 a decrease from 92% (95% CI 90–93) at 0–2 months to 83% (95% CI 81–86) at 7–8 months, from 96% (95% CI 91–98) to 88% (95% CI 82–92) for mRNA-1273 within the first 8 months and from 93% (95% CI 77–98) to 82% (95% CI 78–85) for ChAdOx1-S within the first 6 months after application (Fig 2 black curves, S4 Table in S2 File). Ad26.COV2-S once again exhibits virtually no decline either in the protection against hospitalization starting from 68% (95% CI 60–75) at 2 months to 67% (95% CI 62–72) at 5–6 months, or deaths starting from 68% (95% CI 42–82) and reaching 68% (95% CI 53–78) at 5–6 months (Fig 2, S4 Table in S2 File).

**Fig 3:**
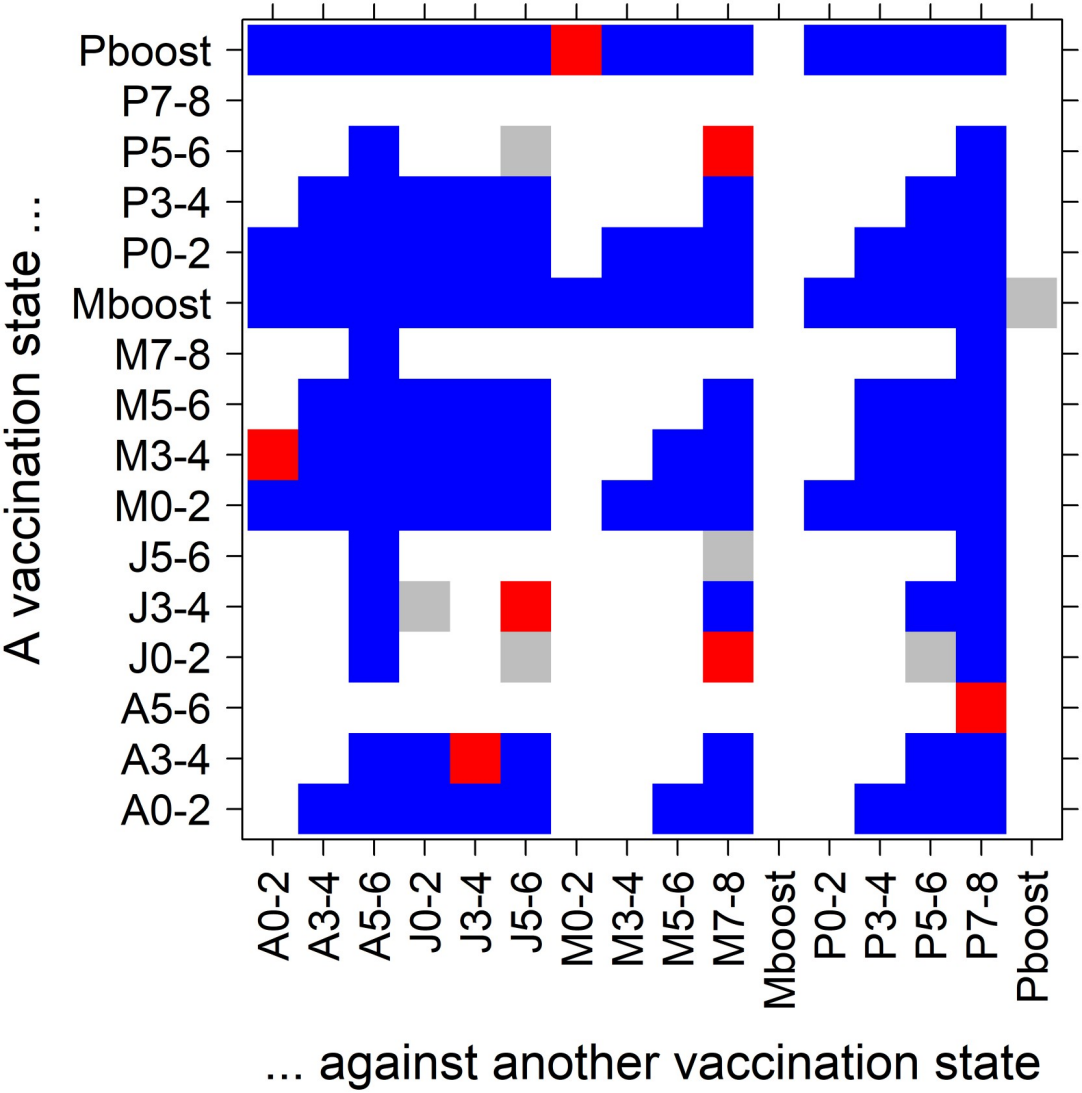
Estimating potential statistical differences between the vaccines. A Z-test has been performed to test for those differences. For each pair of covariates (each covariate is characterized by the vaccine and the time interval since completing the corresponding vaccination scheme), a color is assigned to indicate a degree of statistical significance: blue for 1% (|*Z*| > 2.576), red for 5% (2.576 ≥ |*Z*| > 1.960), and gray for |*Z*| ≤ 1.960. Moreover, only pairs with positive values of the test statistic *Z* are plotted, indicating a positive difference between a respective y-axis covariate and x-axis covariate (values symmetric around the diagonal are negative with the same absolute value). The axis labels are composed of a capital letter (P = BNT162b2 vaccine, M = mRNA-1273 vaccine, A = ChAdOx1-S vaccine, and J = Ad26.COV2-S vaccine) and a number range (months since full vaccination) or ‘boost’ (3rd vaccine dose).

To evaluate the differences between the individual vaccines, we statistically tested whether the corresponding covariates differ significantly; that is, whether their differences are significantly different from zero. To this end, we estimated the distribution of the differences by means of the estimator’s covariance matrix and checked for statistical significance via a Z-test. Fig 3 summarizes the results. We see, for example, that the BNT162b2 booster is quite superior over all other covariates except the mRNA-1273 booster (quite superior to all covariates).

In June-July 2021 the alpha variant of the SARS-CoV-2 virus was largely superseded by the delta variant in Czechia (virus.img.cas.cz/lineages). Therefore, we attempted to disentangle the effects of immunity waning and immunity evasion due to the delta variant on the observed changes in vaccine effectiveness. Evaluating the extra risk of breakthrough infection due to the delta variant, for consistency estimated just for the age cohorts that started to be vaccinated at about the same time, we found a consistent and significant increase in the risk for BNT162b2, mostly significant increase for mRNA-1273 and ChAdOx1-S, and inconclusive results for Ad26.COV2-S (Table 1). Note that these differences do not represent the infection risk increase due to delta; they represent the additional risk increase of a vaccinated individual over the generally higher infectiousness of the delta variant compared to alpha.

**Table 1:** Estimated increase of breakthrough infection hazard ratios (HRs) in times of the SARS-CoV-2 delta variant dominance for age groups having started vaccination in the same month.

Vaccine	March (age 70–80y)		April (age 55–69y)		May (age 35–54y)	
	HR	95% CI	HR	95% CI	HR	95% CI
BNT162b2	1.28	1.09–1.52	1.04	0.95–1.14	1.33	1.27–1.40
mRNA-1273	0.82	0.41–1.67	1.56	1.08–2.25	1.59	1.29–1.98
ChAdOx1-S	1.64	1.05–2.57	1.12	0.74–1.70	1.24	0.82–1.86
Ad26.COV2-S	2.70	0.37–19.63	0.40	0.20–0.78	0.91	0.34–2.43

Regardless of the original vaccine used for the initial vaccination schedule a BNT162b2 booster dose enhances protection against infection to 92% (95% CI 91–92), against hospital admission to 95% (95% CI 94–96), and against death to 97% (95% CI 96–98) (Fig 2). A mRNA-1273 booster dose reaches 93% (95% CI 91–95) protection against infection, 98% (95% CI 95–99) against hospital admission, and close to 100% against death (Fig 2). Combining primary and booster mRNA-based vaccines, boosted effectiveness reached > 91%. The combination of ChAdOx1-S primary and an mRNA booster showed a somewhat lower effectiveness but these estimates are less certain due to a low number of observations (Table 2).

**Table 2:** Vaccine effectiveness against infection after administering the booster vaccine dose for various possible combinations of primary (columns) and booster (rows) vaccines (with the exception of Janssen due to insufficient data). Hazard ratios (HRs) are given.

Vaccine	BNT162b2		mRNA-1273		ChAdOx1-S	
	VE	95% CI	VE	95% CI	VE	95% CI
BNT162b2	0.92	0.91–0.92	0.94	0.91–0.96	0.82	0.68–0.9
mRNA-1273	0.92	0.88–0.95	0.94	0.91–0.95	0.91	0.63–0.98

**Fig 4:**
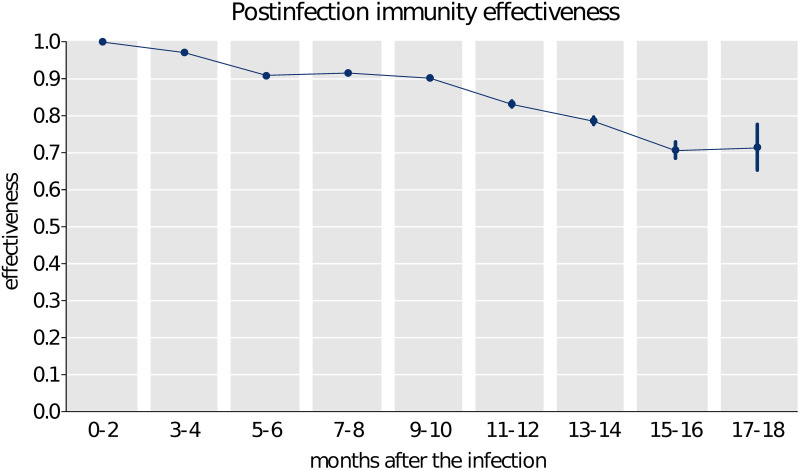
Infection-acquired immunity against reinfection with respect to the delay from the prior infection. The delay 0–2 months is not considered as a new infection which implies 100% effectiveness by definition.

To study reinfections, we used data on PCR-confirmed infections since the beginning of covid-19 pandemic in the Czech Republic; 1,999,315 individuals were infected with SARS-CoV-2 virus until 20th November 2021, of which 12,894 (0.64%) were reinfected. Previous SARS-CoV-2 infection in our population conferred a high and fairly stable level of protection against infection lasting for more than 18 months. In unvaccinated but previously covid-19-positive individuals protection against PCR-confirmed covid-19 infection declined from close to 97% (95% CI 97–97) at 2–4 months through 91% (95% CI 90–91) at 5–6 months down to 83% (95% CI 82–84) at 11–12 months and 72% (95% CI 65–78) at 17–18 months (Fig 4, S5 Table in S2 File).

## Discussion

Our results show a gradual decrease in protective effectiveness of three out of four vaccines used in Czechia to vaccinate against covid-19. The observed decrease was the fastest for protection against infections followed by hospital admissions, while the protection from covid-19-related death was the least affected by the time elapsed from the completion of primary vaccination schedule.

There are several plausible explanations for this decrease and for a corresponding rise in breakthrough infections. One is waning of the immunity conferred by the vaccines, documented for a range of commonly used vaccines and demonstrated for covid-19 vaccines in an increasing number of recent studies [12–14]. The other possibility is the effect of the delta variant, shown to evade to some extent the vaccine-induced immunity [12, 15, 16]. Our sub-analysis of the data suggests but a modest overall effect of the delta variant on the vaccine effectiveness in the studied period. Lacking individual-level information about specific variants causing breakthrough infections we used an indirect method of time dummies corresponding to the period of delta variant dominance. Since this approach may be affected by co-linearity (“influence” of the absolute time will be mismatched with the waning), we present these estimates just as secondary results; the primary effectiveness estimates are averaged over the viral variants. However, it is likely that long-term estimates of vaccine effectiveness correspond to the period of dominating delta variant. This is not an issue for boosters, which have been given only after delta reached overwhelming dominance.

A largely unstudied factor that could affect the observed vaccine effectiveness are changes in behaviour of vaccinated vs. unvaccinated persons and a possible effect of infection control measures due to differential access of the unvaccinated individuals to many social activities or due to differential testing strategies, since vaccinated people and people within 6 months after their PCR-positivity have not been required to undergo testing as often as the others. Indeed, analyses of vaccine effectiveness and its temporal dynamics generally assume that both vaccinated and unvaccinated persons behave similarly and we assume this in our study as well. This possible limitation of this study is applicable mainly to the endpoint of confirmed infection and to the lesser extent to the hospitalisation or death endpoints.

The one-dose Janssen vaccine appears in our analysis to defy the general trend of protection decay. While it starts at a significantly lower effectiveness, it holds it over the all 6 months we consider. To our knowledge, this somewhat counter-intuitive result has not yet been reported and as such is not easy to interpret. However, since this vaccine was introduced to the Czech Republic much later than the other three vaccines and only one dose is required for complete vaccination, it is plausible that this vaccine was mostly chosen by people with different social and behavioural characteristics compared to the two-dose vaccines. Since we cannot support this suggestion with data, we leave this as a suggestion for further studies.

We show that administration of booster doses of two approved mRNA vaccines brings the observed effectiveness to above 90% for infections, hospital admissions and deaths alike. Booster doses are highly efficient for preventing serious or fatal infections. Although our results are in a general agreement with the study on protective effect of vaccine booster in Israel [17], we cover a more extensive period of booster applications, use of the mRNA-1273 vaccine as a booster, and do not limit ourselves to any specific age group.

Protection afforded by previous covid-19 infection declines over time, too, but at a slower rate compared to the post-vaccination immunity. Whereas several studies consistently report that protection against reinfection declines [7–10], we are the first to describe the long-term temporal dynamics of infection-induced immunity against SARS-CoV-2 reinfections. We note that this finding relates only to directly confirmed primary infections (possibly associated with test-seeking behaviour and severity of the disease) and may not be translatable to evidence of previous infection from antibody testing.

In this study we used a Cox model with calendar time, which has an obvious advantage—our results are independent of factors that influence the risk of all the subjects equally, such as a change in the basic reproduction number, viral prevalence in the population, non-pharmaceutical interventions, weather or seasonal influences, or a dominant virus variant—all these factors can be included in the baseline hazard function of the Cox model. All this makes our findings comparable with and transferable to other contexts. Indeed, similar studies have come to similar conclusions [12]. Our results are also robust with respect to under-reporting provided the reporting rate is the same for all subjects, because then the Cox regression equation is only multiplied by a constant, so the estimation of the HRs remains correct.

The model, however, has some limitations. Importantly, the dependence of individual hazard function on covariates may be non-log-linear. This happens, for instance, when the detection rate depends on a characteristic of a subject—e.g. unvaccinated are being tested more often than the vaccinated. If this were true the vaccine effectiveness would be overestimated, yet the estimates of the HR increase (i.e. VE decline) over time would still be valid (provided that the testing propensity does not change in time). Equally such a case could arise if the vaccinated behaved more riskily than the unvaccinated—the effectiveness then would be underestimated, yet the estimates of the relative increase of HR (and the consequent decrease of VE) would again be valid provided that this behaviour does not change substantially over the study period. It is also worth noting that unlike infections the hospitalization and mortality data are less likely to suffer from the aforementioned bias as the facts of hospitalizations and deaths depend much less on test seeking behavior.

In addition, as everywhere, only a certain fraction of infections are reported and hence the results could possibly be distorted by this fact; that fraction of unreported infections is called the ascertainment rate. This potential distortion is less severe for the estimates of VE if the ascertainment rate is comparable in the group of the vaccinated individuals and in the control group of the unvaccinated virgin population, since biases then cancel out. However, the problem could be more serious for the estimates of post-infection immunity protection; here, even if the ascertainment rates were comparable in the group of once infected and in the control group of covid-19 virgins, there would be an undetected part of formerly infected and hence protected in the control group. Therefore, the risk of infection in the truly virgin population would be underestimated, while the estimate of the infection risk in the treatment group would not be biased, as all have been infected here. As a consequence, the protection by the past infection is likely underestimated. Determining size of this possible bias is complicated e.g. by the fact that the unreported cases are more likely to be mild ones, providing less protection.

Concerns may arise as to what extent are the results affected by the changing environment due to such aspects as weather, counter-epidemic measures, people’s behavior, etc. Thanks to the fact that we use calendar instead of relative time in our analyses, these changes are handled by the baseline hazard function, provided that the environment affects the “treatment” groups in the same way as the “control” group. This can be the case for both the weather (changing the amount of time spent indoors) and the overall counter-epidemic measures (reducing the number of risk contacts). On the other hand, as already emphasized above, differences in behavioral effects cannot be fully excluded (vaccinated or even unvaccinated people may behave in riskier ways); thus, the VE should be understood including the potential behavioral responses. Also, it should be stressed that the fact that the environmental effects “cancel out” in the Cox model with calendar time (the results do not depend on the baseline hazard in any way) effectively precludes studying these effects by means of this model.

## Conclusion

We used a comprehensive national population-based database containing individual level data about all detected SARS-CoV-2 infection cases to estimate many important characteristics of the post-vaccination and post-infection immunity in the population of the Czech Republic, covering all four vaccines currently approved in the EU and the protection from infection, hospital admission and death. The results strongly advocate for a timely and widespread administration of the third (i.e., booster) dose. Covid-19 will undoubtedly continue to disrupt everyday lives and cause suffering and loss of life around the globe and vaccine effectiveness data such as the ones presented in this study can bring an important insight for policy makers in order to limit the worst impacts of the current pandemic.

## Supporting information

S1 File More information on the use of the Cox regression method. Details on the statistical model used to process infection and vaccination data and to produce the main results of this study.
(PDF)

S2 File Supporting Figures and Tables. S1-S3 Figs and S1-S5 Tables that are referred to within the main text. S6-S10 Tables indicating numbers of respective cases behind results plotted in Figs 2 and 4 in the main text.
(PDF)

S3 File Detailed age- and gender-structured data on temporal dynamics of vaccination and infection in the Czech Republic during the study period. Data presented on a weekly basis, with the date in the first column indicating the middle of the respective week (week is not a calendar week, but from Saturday till Friday, since vaccination started on Saturday, December 26, 2020).
(ODS)
