# Correction: Protection provided by vaccination, booster doses and previous infection against covid-19 infection, hospitalisation or death over time in Czechia

**DOI:** 10.1371/journal.pone.0295159

**Published:** 2023-11-28

**Authors:** The PLOS ONE Staff

The publisher apologizes for the error. There is an error in affiliation 11 for author Jan Trnka. The correct affiliation 11 is: Department of Biochemistry, Cell and Molecular Biology, Third Faculty of Medicine, Charles University, Rusk´a 87, 10000 Praha 10, Czech Republic.
