# Supplementary material for: Protection provided by vaccination, booster doses and previous infection against covid-19 infection, hospitalisation or death over time in Czechia
Source: PLoS One. 2022 Jul 8;17(7):e0270801. doi: 10.1371/journal.pone.0270801 (PMC9269461; doi:10.1371/journal.pone.0270801)
Supplement: S1 File — Details on the statistical model used to process infection and vaccination data and to produce the main results of this study. (PDF) [file pone.0270801.s001.pdf]

Protection provided by vaccination, booster doses and previous infection against covid-19 infection, hospitalisation or death over time in Czechia

Supporting Information, S1 File: Methods

A Cox regression with time-varying covariates

We use a Cox proportional Hazard model with time varying covariates. In the model, we let each individual to go through the several "vaccination" (covariate `VaccStatus`) and "post-infection" (`InfPrior`) states. The outcomes (events) are either (a confirmed) infection (`Infected`, may be repeated), hospitalization (`Hospitalized`), or death of covid (`DeadByCov`). Deaths of other reasons are also recorded (`DeadByOther`), leading to withdrawal from the study at the time of the event. Fixed (non-time-dependent) covariates include sex (`Sex`) and age category (`AgeGr`). The input for the Cox regression model (`coxph` from `survival` `R` package) consists of one or more records for each subject, each referring to an interval from `T1` to `T2`, containing the values of covariates $InfPrior$, $VaccStatus$, $AgeGr$, Sex valid in $[T1, T2)$ and indicators of outcomes `Infected, Hospitalized, DeadByCov, DeadByOther`, happening at $T2$. There may be (and typically is) several records for each subject, each corresponding to a time interval in which the covariates are constant and in the interor of which no events happen.

Time is measured in days and we take the day before vaccination started (Dec 26th, 2021) as time zero in all analyses except for the reinfection analysis in which we take May 1st, 2020 (two months after the first cases) as time zero. The `VaccStatus` categorical covariate may take the following values:

`_Unvacc`: The subject is not vaccinated (this value is taken as reference).

`V_first1` The subject is from 14 to $14 + 61 - 1 = 74$ days after the first dose of vaccine `V` but not 14 days or more after a second dose. `V` may be `A`–ChAdOx1-S, `M`–mRNA-1273 or `P`–BNT162b2.

`V_first2plus`: The subject is 75 days or more after the first dose of vaccine `V` but not 14 days or more after a second dose.

`VX`: The subject is between $14 + (X - 1) * 61$ and $14 + (X) * 61 - 1$ days after the final dose of vaccine `V` but not 7 days or more after a booster. In addition to `P/M/A`, the vaccine may be also `J` (Ad26.COV2-S).

`V`**`boost:`** The subject is 7 days or more after a booster by vaccine `V`.

The `InfPrior` may take the following values:

`_None:` The subject has not been infected previously.

`X`**`:`** The subject is from $(X-1) \times p$ to $X \times p - 1$ days after the last positive test for covid, where $p = 61$ in the analyses of reinfections an $p = 91$ in the remaining analyses.

`rest:` In reinfection analysis: the subject is $9 \times 61 = 549$ days or more after the last positive test, in the remaining analyses: the subject is $3 \times 91 = 273$ days or more after the last positive test.

By default, for each subject, the intervals cover the entire period from 26th. December 2020 to 20. November 2021. The period is shortened if either

- The subject is reported to die
- The subject is reported to obtain booster by ChAdOx1-S or Ad26.COV2-S
- The subject is $4 \times 61 + 14 = 258$ days after the final dose and has not yet obtained a booster.
- The subject is hospitalized (only in the hospitalization analysis)
- The subject is infected or gets a vaccine (only in the reinfection analysis)

For better understanding, here we show a complete data record of four sample subjects (A0,A1,A2,A3) determined to the infection analysis. All of them are recorded from T=0 (26.12.2020) until T=314 (4.11.2021).

A0 is not vaccinated and gets infected at the last day of the study. A1 has not been infected before, but gets infected (Day 140) and dies of covid-19 (Day 150) before being vaccinated. A2 became first-dose vaccinated with BNT162b2 on day 114, was infected between the first and second dose (day 142), got the second dose (day 220) and survives until the end of the study. A3 has been infected 20 days before beginning of the study, gets vaccinated by Ad26.COV2-S (Day 150) and is not infected until the end.

The input of `coxph` routine is displayed in Table 1. Note that there is typically more records then events as each follow-up covariate has to have its own interval. Table 2 gives details on the performed analyses.

Table 1: Sample input to Cox regression.

Subject	T1	T2	Infected	DeadCovid	DeadOther	InfPrior	VaccStatus	AgeGr	Sex
A0	0	313	1	0	0	_none	_unvacc	40-44	F
A0	313	314	0	0	0	1	_unvacc	40-44	F
A1	0	140	1	0	0	_none	_unvacc	75-79	F
A1	140	150	0	1	0	_none	_unvacc	75-79	F
A2	0	128(=114+14)	0	0	0	_none	_unvacc	45-49	M
A2	128	142	1	0	0	_none	P_first1	45-49	M
A2	142	189(=128+61)	0	0	0	1	P_first1	45-49	M
A2	189	220	0	0	0	1	P_first2plus	45-49	M
A2	220	233(=142+91)	0	0	0	1	P1	45-49	M
A2	233	281(=220+61)	0	0	0	2	P1	45-49	M
A2	281	314	0	0	0	2	P2	45-49	M
A3	0	71(=0-20+91)	0	0	0	1	_unvacc	40-44	F
A3	71	162(=71+91)	0	0	0	2	_unvacc	40-44	F
A3	162	164(=150+14)	0	0	0	3	_unvacc	40-44	F
A3	164	225(=164+61)	0	0	0	3	J1	40-44	F
A3	225	253(=162+91)	0	0	0	3	J2	40-44	F
A3	253	286(=225+61)	0	0	0	rest	J2	40-44	F
A3	286	314	0	0	0	rest	J3	40-44	F

Table 2: Details on analyses. XDeltaInf – a dummy equal to one if the VaccStatus value corresponds to vaccine X and the interval $T1 \geq$ Jul-01-2021, * – 61 days periods (otherwise 91 day periods for InfPrior).

Analysis	Ages	Event	Covariates
Infections	all	Infected	InfPrior, VaccStatus, Sex, AgeGr
Reinfections	all	Infected	InfPrior*, Sex, AgeGr
Hospitalizations	all	Hospitalized	VaccStatus, Sex, AgeGr
Deaths	all	DeadByCov	VaccStatus, Sex, AgeGr
Boosters	all	Infected	InfPrior, VaccStatus, Sex, AgeGr
Delta March	70–79	Infected	InfPrior, VaccStatus, Sex, AgeGr, ADeltaInf, JDeltaInf, MDeltaInf, PDeltaInf
Delta April	55–69	Infected	dtto.
Delta May	30–54	Infected	dtto.
