# Supplementary material for: Protection provided by vaccination, booster doses and previous infection against covid-19 infection, hospitalisation or death over time in Czechia
Source: PLoS One. 2022 Jul 8;17(7):e0270801. doi: 10.1371/journal.pone.0270801 (PMC9269461; doi:10.1371/journal.pone.0270801)
Supplement: S2 File — S1-S3 Figs and S1-S5 Tables that are referred to within the main text. S6-S10 Tables indicating numbers of respective cases behind results plotted in Figs 2 and 4 in the main text. (PDF) [file pone.0270801.s002.pdf]

Protection provided by vaccination, booster doses and previous infection against covid-19 infection, hospitalisation or death over time in Czechia

Supporting Information, S2 File: Figures and Tables

Table S1: Vaccination timeline according to the age and professional prioritizations in the Czech Republic.

Age category	Vaccination start	Professional and other categories	Vaccination start
80+	2021-01-15	critical infrastructure	2020-12-27
70+	2021-03-01	health-care workers	2021-01-26
65+	2021-04-14	pedagogical staff	2021-02-27
60+	2021-04-23	persons with chronic disease 1. priority	2021-03-24
55+	2021-04-28	persons with chronic disease 2. priority	2021-04-12
50+	2021-05-05	social workers	2021-04-07
45+	2021-05-11	academic staff	2021-05-03
40+	2021-05-17	foreigners	2021-06-11
35+	2021-05-24		
30+	2021-05-26		
16+	2021-06-04	boosters for prioritized	2021-09-02
12+	2021-07-01	boosters	2021-09-20

Figure S1: Daily incidence of covid-19 in the Czech Republic. Time ranges from March 1, 2020 till November 20, 2021, and daily incidence is stratified by age, per 100,000 individuals in the respective age groups.

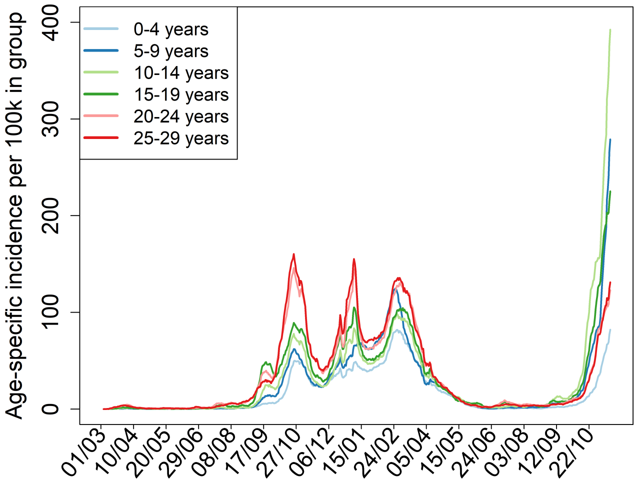

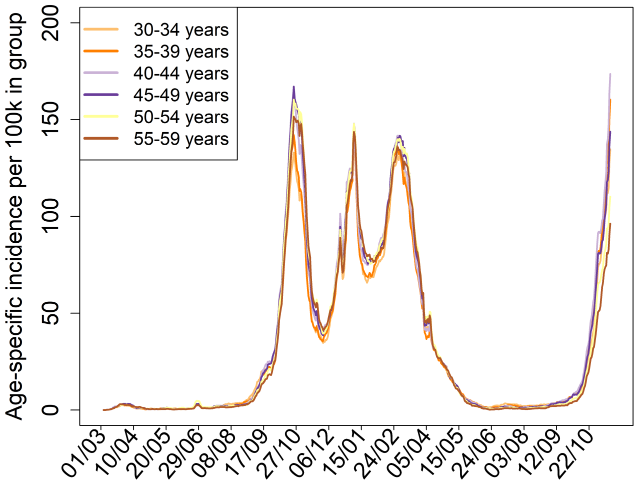

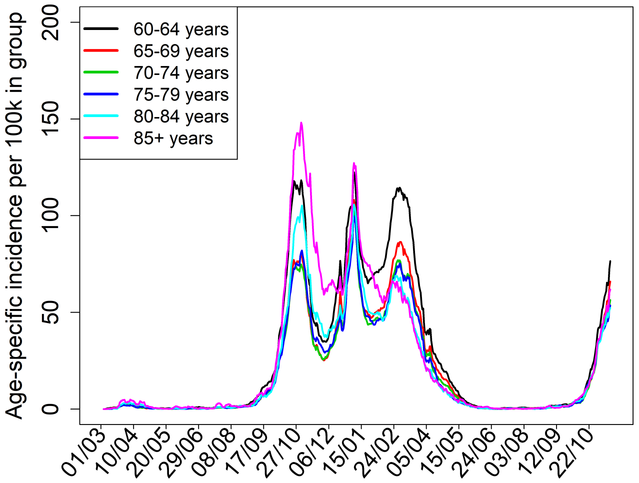

Table S2: Descriptive statistics: population and epidemic characteristics in the Czech Republic; ISID = the Czech National Information System of Infectious Diseases.

	Dataset				Population by 26/12/2020			Events from 26/12/2020			
Age group	Number	Data errors	Dead by 26/12/2020 covid-19	other	From ISID	Added	Total	Infec-ted	Hospi-talized	Dead covid-19	Dead other
0-11	203190	7	0	3	203180	1163925	1367105	149584	699	4	9
12-15	258888	8	0	0	258880	197608	456488	73379	247	0	6
16-17	142448	2	1	2	142443	52573	195016	29746	158	1	4
18-24	493435	17	1	5	493412	176080	669492	92660	696	4	31
25-29	405012	10	7	8	404987	215938	620925	83115	1008	14	38
30-34	479206	9	12	13	479172	239759	718931	96774	1655	32	59
35-39	520232	8	23	10	520191	233119	753310	104362	1941	47	93
40-44	657552	21	47	18	657466	235855	893321	127504	3082	93	178
45-49	709138	15	66	53	709004	173582	882586	133859	4665	210	336
50-54	557757	11	106	67	557573	133510	691083	99162	5004	316	452
55-59	547417	8	237	125	547047	122686	669733	90846	6452	583	673
60-64	513110	16	432	200	512462	113003	625465	68935	8224	1130	1028
65-69	578574	20	892	333	577329	95089	672418	58480	11500	2212	1824
70-74	546312	20	1663	657	543972	77205	621177	50058	14548	3559	2840
75-79	396614	24	2156	800	393634	23567	417201	35890	14372	4171	3225
80+	420199	20	5723	2739	411717	35809	447526	40701	21986	8433	9150
Total	7429084	216	11366	5033	7412469	3289308	10701777	1335055	96237	20809	19946
Errors	No age	8491	No sex	343							

Figure S2: Cumulative number of infected individuals, severe cases and deaths in different age groups from December 26, 2020 till November 20, 2021 (time span of the study). This figure vizualizes some information of Table S2.

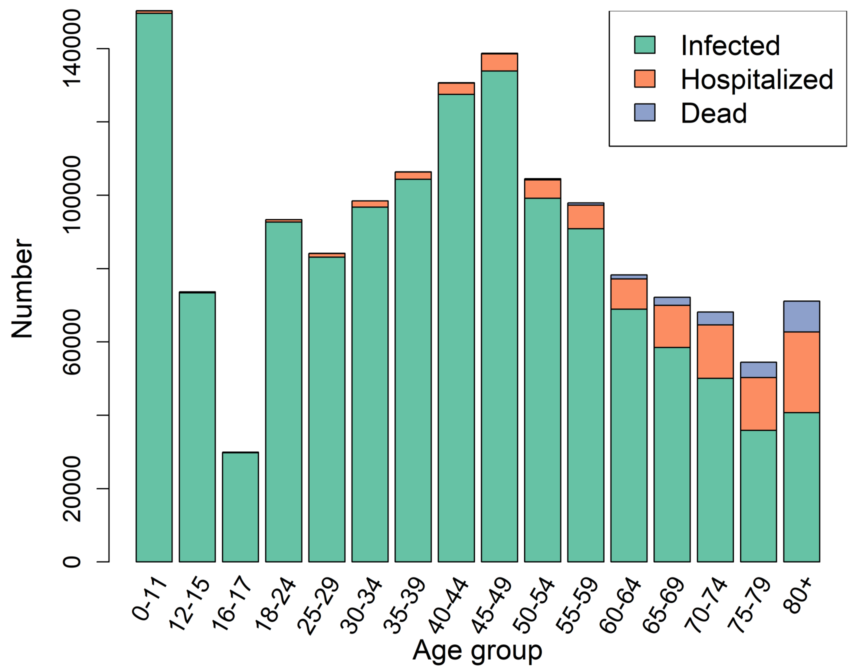

Table S3: Descriptive statistics: vaccine distribution among different age groups until November 20, 2021.

Age	Completed vaccination					Booster doses				
group	BNT162b2	mRNA-1273	ChAdOx1-S	Ad26.COV2-S	Total	BNT162b2	mRNA-1273	ChAdOx1-S	Ad26.COV2-S	Total
0-11	1435	27	2	4	1468	1	0	0	0	1
12-15	157523	2837	2	1	160363	7	1	0	0	8
16-17	105280	2249	39	306	107874	100	14	0	0	114
18-24	333076	22399	2446	39579	397500	4619	412	0	2	5033
25-29	261001	21720	3443	31346	317510	7999	695	2	5	8701
30-34	323970	24252	4864	32263	385349	9896	1039	4	5	10944
35-39	364023	27941	7219	33093	432276	14676	1617	2	10	16305
40-44	481994	36869	12470	37326	568659	23792	2580	13	19	26404
45-49	529313	40118	16480	39653	625564	30824	3384	7	17	34232
50-54	415101	34596	18013	31276	498986	28330	3255	9	19	31613
55-59	407622	34850	25000	29712	497184	32840	3742	10	24	36616
60-64	380452	34934	35010	27317	477713	31320	3758	5	24	35107
65-69	413874	44337	63135	27171	548517	33700	4876	11	31	38618
70-74	338152	56931	103284	19660	518027	110182	15891	35	56	126164
75-79	234084	45325	81112	11940	372461	125067	16403	24	62	141556
80+	264215	40220	64056	9414	377905	163649	18402	54	58	182163
Total	5011115	469605	436575	370061	6287356	617002	76069	176	332	693579

Figure S3: Cumulative number of individuals vaccinated by various vaccines, structured by age, from December 26, 2020 till November 20, 2021 (time span of the study). This figure vizualizes some information of Table S3. Legend: P2 – two doses of BNT162b2, M2 – two doses of mRNA-1273, A2 – two doses of ChAdOx1-S, J1 – one dose of Ad26.COV2-S, P3 – three doses of BNT162b2, M3 – three doses of mRNA-1273.

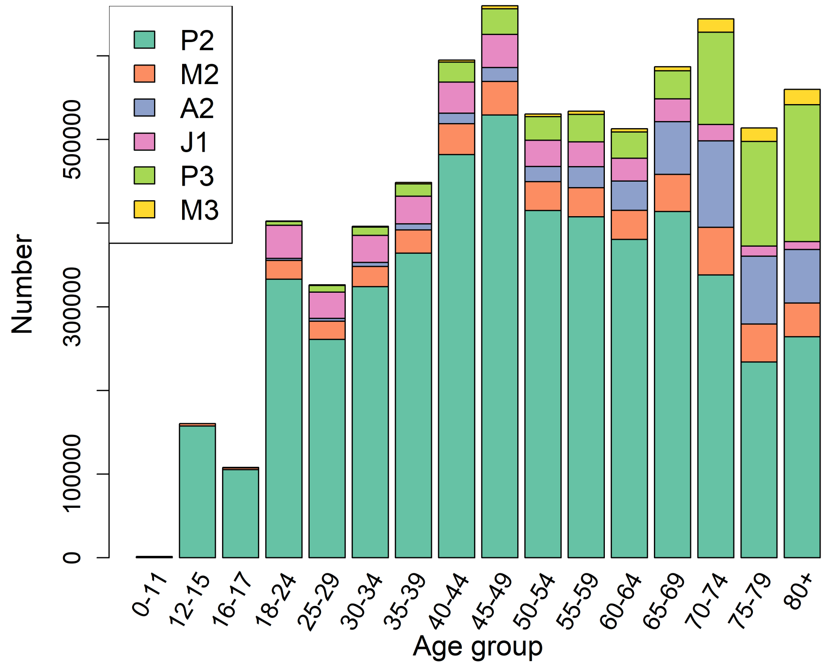

Table S4: Model outcomes for the effect of vaccination against infection, hospitalization and death. Hazard ratios together with 95% confidence intervals are provided. See the accompanying text for the meaning of model covariates.

Model covariate	Infections	Hospitalizations	Deaths
AgeGr0-11	0.79 (0.78, 0.8)	0.01 (0.01, 0.01)	0 (0, 0)
AgeGr12-15	1.33 (1.32, 1.35)	0.01 (0.01, 0.01)	0 (0, 0)
AgeGr16-17	1.37 (1.35, 1.39)	0.01 (0.01, 0.01)	0 (0, 0)
AgeGr18-24	1.28 (1.26, 1.29)	0.01 (0.01, 0.01)	0 (0, 0)
AgeGr25-29	1.21 (1.19, 1.22)	0.02 (0.02, 0.02)	0 (0, 0)
AgeGr30-34	1.21 (1.2, 1.23)	0.03 (0.03, 0.03)	0 (0, 0)
AgeGr35-39	1.27 (1.25, 1.28)	0.03 (0.03, 0.04)	0 (0, 0)
AgeGr40-44	1.35 (1.33, 1.36)	0.05 (0.05, 0.05)	0 (0, 0)
AgeGr45-49	1.49 (1.48, 1.51)	0.07 (0.07, 0.08)	0.01 (0.01, 0.01)
AgeGr50-54	1.41 (1.4, 1.43)	0.1 (0.1, 0.11)	0.02 (0.01, 0.02)
AgeGr55-59	1.33 (1.32, 1.35)	0.14 (0.14, 0.14)	0.03 (0.03, 0.03)
AgeGr60-64	1.05 (1.04, 1.07)	0.19 (0.19, 0.2)	0.06 (0.06, 0.07)
AgeGr65-69	0.82 (0.81, 0.83)	0.26 (0.25, 0.26)	0.12 (0.11, 0.12)
AgeGr70-74	0.76 (0.75, 0.77)	0.37 (0.36, 0.38)	0.22 (0.21, 0.23)
AgeGr75-79	0.85 (0.83, 0.86)	0.58 (0.57, 0.6)	0.42 (0.4, 0.43)
SexZ	1.06 (1.05, 1.06)	0.69 (0.68, 0.69)	0.47 (0.46, 0.49)
VaccStatusA_first1	0.64 (0.62, 0.67)	0.42 (0.39, 0.45)	0.36 (0.31, 0.4)
VaccStatusA_first2plus	0.39 (0.34, 0.44)	0.34 (0.27, 0.44)	0.34 (0.22, 0.53)
VaccStatusA1	0.17 (0.15, 0.2)	0.13 (0.09, 0.19)	0.07 (0.02, 0.23)
VaccStatusA2	0.28 (0.27, 0.29)	0.23 (0.2, 0.25)	0.13 (0.1, 0.18)
VaccStatusA3	0.45 (0.44, 0.46)	0.3 (0.28, 0.32)	0.18 (0.15, 0.22)
VaccStatusA4	0.41 (0.3, 0.55)	0.3 (0.14, 0.63)	0.32 (0.08, 1.29)
VaccStatusJ1	0.32 (0.3, 0.34)	0.32 (0.25, 0.4)	0.32 (0.18, 0.58)
VaccStatusJ2	0.3 (0.29, 0.32)	0.26 (0.22, 0.32)	0.25 (0.15, 0.42)
VaccStatusJ3	0.33 (0.31, 0.35)	0.33 (0.28, 0.38)	0.33 (0.22, 0.48)
VaccStatusJ4	0.37 (0.28, 0.50)	0.19 (0.07, 0.51)	0.20 (0.03, 1.40)
VaccStatusM_first1	0.34 (0.32, 0.37)	0.35 (0.31, 0.40)	0.31 (0.26, 0.39)
VaccStatusM_first2plus	0.23 (0.18, 0.3)	0.21 (0.1, 0.43)	0.24 (0.06, 0.96)
VaccStatusM1	0.1 (0.09, 0.11)	0.06 (0.04, 0.08)	0.05 (0.02, 0.09)
VaccStatusM2	0.15 (0.14, 0.15)	0.1 (0.08, 0.13)	0.04 (0.01, 0.1)
VaccStatusM3	0.22 (0.21, 0.23)	0.14 (0.12, 0.16)	0.09 (0.06, 0.14)
VaccStatusM4	0.35 (0.33, 0.37)	0.19 (0.16, 0.22)	0.12 (0.08, 0.18)
VaccStatusMboost	0.07 (0.05, 0.09)	0.02 (0.01, 0.05)	0 (0, 0)
VaccStatusP_first1	0.52 (0.51, 0.53)	0.39 (0.37, 0.41)	0.38 (0.35, 0.41)
VaccStatusP_first2plus	0.4 (0.37, 0.43)	0.33 (0.25, 0.43)	0.22 (0.11, 0.42)
VaccStatusP1	0.13 (0.13, 0.14)	0.1 (0.09, 0.11)	0.08 (0.07, 0.1)
VaccStatusP2	0.26 (0.25, 0.26)	0.09 (0.08, 0.1)	0.07 (0.06, 0.1)
VaccStatusP3	0.32 (0.32, 0.33)	0.14 (0.13, 0.15)	0.1 (0.08, 0.12)
VaccStatusP4	0.47 (0.46, 0.48)	0.25 (0.24, 0.27)	0.17 (0.14, 0.19)
VaccStatusPboost	0.08 (0.08, 0.09)	0.05 (0.04, 0.06)	0.03 (0.02, 0.04)
InfPrior1	0 (0, 0)		
InfPrior2	0.09 (0.09, 0.09)		
InfPrior3	0.09 (0.09, 0.09)		
InfPriorrest	0.16 (0.16, 0.17)		

Table S5: Infection-acquired immunity against reinfection.

Model covariate	Hazard ratio
AgeGr0-11	0.6 (0.6, 0.61)
AgeGr12-15	0.99 (0.98, 1)
AgeGr16-17	1.05 (1.03, 1.06)
AgeGr18-24	1.1 (1.09, 1.11)
AgeGr25-29	1.06 (1.05, 1.07)
AgeGr30-34	1.01 (1, 1.02)
AgeGr35-39	1.03 (1.02, 1.04)
AgeGr40-44	1.11 (1.1, 1.12)
AgeGr45-49	1.24 (1.23, 1.25)
AgeGr50-54	1.21 (1.2, 1.22)
AgeGr55-59	1.15 (1.14, 1.16)
AgeGr60-64	0.89 (0.88, 0.9)
AgeGr65-69	0.67 (0.66, 0.68)
AgeGr70-74	0.64 (0.63, 0.65)
AgeGr75-79	0.72 (0.71, 0.73)
InfPrior1	0 (0, 0)
InfPrior2	0.03 (0.03, 0.03)
InfPrior3	0.09 (0.09, 0.1)
InfPrior4	0.08 (0.08, 0.09)
InfPrior5	0.1 (0.09, 0.1)
InfPrior6	0.17 (0.16, 0.18)
InfPrior7	0.21 (0.2, 0.22)
InfPrior8	0.29 (0.27, 0.32)
InfPrior9	0.28 (0.22, 0.35)
InfPriorrest	0.16 (0.12, 0.21)
SexZ	1.06 (1.06, 1.06)

Numbers behind Figures 2 and 3 in the main text

Here we present how many individuals were infected, got a severe disease or died in different vaccine groups and time ranges since completing vaccination, corresponding to individual ‘dots’ in Fig. 2 in the main text.

Table S6: Numbers of individuals who were infected, got a severe disease or died after infection when vaccinated by BNT162b2 (Pfizer/BioNTech) and which entered our analyses.

	0-2 months	3-4 months	5-6 months	7-8 months	booster
Infection	5986	34457	24152	10669	746
Hospitalization	599	527	1134	1351	106
Death	180	54	121	212	16

Table S7: Numbers of individuals who were infected, got a severe disease or died after infection when vaccinated by mRNA-1273 (Moderna) and which entered our analyses.

	0-2 months	3-4 months	5-6 months	7-8 months	booster
Infection	287	1461	1729	1335	60
Hospitalization	34	71	171	160	4
Death	9	4	20	26	0

Table S8: Numbers of individuals who were infected, got a severe disease or died after infection when vaccinated by ChAdOx-1-S (AstraZeneca) and which entered our analyses.

	0-2 months	3-4 months	5-6 months
Infection	188	2451	5289
Hospitalization	27	402	834
Death	3	43	121

Table S9: Numbers of individuals who were infected, got a severe disease or died after infection when vaccinated by Ad26.COV2-S (Johnson&Johnson) and which entered our analyses.

	0-2 months	3-4 months	5-6 months
Infection	1418	1739	1278
Hospitalization	79	105	147
Death	11	15	27

Here we present how many individuals were reinfected in different time ranges since previous (first) infection, corresponding to individual 'dots' in Fig. 3 in the main text.

Table S10: Numbers of individuals who were reinfected after (first) infection and which entered our analyses.

0-2 months	3-4 months	5-6 months	7-8 months	9-10 months
5	1380	2590	1117	1564

11-12 months	13-14 months	15-16 months	17-18 months
1654	1747	670	79
